# Does heterozygosity at the *DMRT3* gene make French trotters better racers?

**DOI:** 10.1186/s12711-015-0095-7

**Published:** 2015-02-26

**Authors:** Anne Ricard

**Affiliations:** INRA, UMR 1313 Génétique Animale et Biologie Intégrative, 78352 Jouy-en-Josas, France; IFCE, Recherche et Innovation, 61310 Exmes, France

## Abstract

**Background:**

Recently, a mutation was discovered in the *DMRT3* gene that controls pacing in horses. The mutant allele A is fixed in the American Standardbred trotter breed, while in the French trotter breed, the frequency of the wild-type allele C is still 24%. This study aimed at measuring the effect of *DMRT3* genotypes on the performance of French trotters and explaining why the polymorphism still occurs in this breed. Using a mixed animal model, genetic parameters and environmental effects on performance traits were estimated from data on 173 176 French trotter races. The effect of the *DMRT3* gene was then estimated by the effect of genotype at the highly linked SNP *BIEC2-620109* (C-C, A-T) for 630 horses. A selection scheme that included qualification and racing performances was modeled to (1) verify if the observed superiority of heterozygous CT horses at this SNP could be explained only by selection and (2) understand why allele C has not disappeared in French trotters.

**Results:**

Heritability of racing performance traits was high for qualification test (0.56), moderate for annual earnings per finished race (0.26 to 0.31) and low for proportion of disqualified races (0.06 to 0.09). Genotype CC was always unfavorable compared to genotype TT for qualification: the probability to be qualified was 20% for CC *vs.* 48% for TT and earnings were -0.96 *σ*_*y*_ lower for CC than for TT. Genotype CT was also unfavorable for qualification (40%) and earnings at 3 years (-0.21 *σ*_*y*_), but favorable for earnings at ages greater than 5 years: +0.41 *σ*_*y*_ (*P* = 7.10^−4^). Selection on qualification could not explain more than 19% of the difference between genotypes CC and CT in earnings at ages greater than 5 years. Only a scenario for which genotype CT has a favorable effect on the performance of horses older than 5 years could explain that the polymorphism at the *DMRT3* gene still exists in the French trotter breed.

**Conclusions:**

The use of mature horses in the French racing circuit can explain that the CA genotype is still present in the French trotter horses.

## Background

In 2012, Andersson *et al*. [1] reported a mutation in the *DMRT3* (*doublesex and mab-3 related transcription factor 3*) gene that affects locomotion in horses. The role of this gene on a specific subset of spinal cord neurons was demonstrated in mice. A single base change (A/C) at nucleotide position 22999655 on horse chromosome 23 causes a premature stop codon and results in a truncated protein. The *DMRT3* gene was first located by a GWAS (genome-wide association study) in the small Icelandic horse breed in which all horses with pacing ability, except one, were homozygous AA, while only 31% of the horses without pacing ability were homozygous AA. Allele A is fixed in most breeds that have been selected for trot racing or for pace racing, such as the American Standardbred [2], whereas allele C is fixed in breeds that have been selected for gallop racing, such as the Thoroughbred (high-speed galloping breed), the Arab (endurance riding and show breed) and Swedish warmblood horses [2]. In the original publication [1], the Swedish team also genotyped 47 French trotters and found a frequency of only 77% for allele A in this breed. Recently, this result was confirmed by Promerová *et al.* [2]. However, the negative effect of allele C on the performance of trotter horses was clearly demonstrated by comparing the estimated breeding values for racing performances and earnings of Swedish Standardbred horses including crossbred imported French trotters (AC carriers (n = 17) and AA carriers (n = 206)). Hence, the following paradox: if allele C of the *DMRT3* gene has an adverse effect only on trotting ability, why is it still present in the French trotter population which has been under selection for racing performance for over a century?

In this section, we briefly introduce the life of a trotter horse for readers unfamiliar with horse racing. For trotter horses to take part in races, first, they must pass a “qualification” test. This consists of a timed event which is organized in a similar manner to a race over a distance of 2000 meters. The qualifying time for the 2000-meter race depends on the age of the candidate (from age 2 years) and has been modified over the years as the performance of racing horses has improved. Approximately 40% of the horses of a given generation are qualified. Once French trotters are qualified for racing, their career consists of three stages [3]. The first stage, although not common practice, starts at the age of 2 years (20% of 3-year-old racers start at the age of 2 years). The second stage, between 3 and 4 years of age, is the period when a trotter reaches the peak of its racing career. Finally, only the best horses enter the third stage and continue racing between 5 and 10 years of age. One third of the horses stop racing in their fourth year and the same proportion of horses stop in each of the following years, so only a very few horses still race at 9 or 10 years of age. In trotting races, the first seven finishers receive prize money at each race, which decreases by about 50% per place. The next nine fastest finishers are ranked, whereas the slowest horses are not ranked. Horses that break stride are disqualified. Trotting races are usually harness races, a form of horse racing in which the horse pulls an ultra-light roadster called a sulky. However, in France, a specific type of trotting race is organized called racing under saddle (“trot monté”), in which the jockey rides on the horse’s back.

Thus, the aims of this study were to (1) measure the effect of *DMRT3* genotypes on various traits associated with the performance of trotter horses in France, (2) explain why the polymorphism for this gene is still present in the French trotter breed (in French “Trotteur Français”), and (3) determine whether it is possible to predict when allele C might disappear from the population of trotter horses that are currently under selection in France.

## Methods

### Performance data

All annual results for trotters that took part in French races from 1996 to 2011 were provided by the Society for the Promotion of French Horse Breeding (SECF, Société d’encouragement à l’Elevage du Cheval Français), the parent corporation for trotting races in France. The following data were available for each horse for each year for both harness racing and racing under saddle: annual earnings, number of races entered and number of times a horse was disqualified. An additional dataset containing qualification dates for all qualified horses and the best-recorded time per kilometer was provided by the French Institute for Horses and Riding (IFCE, Institut Français du Cheval et de l’Equitation). Pedigrees were also provided by the IFCE.

Considering that a horse can race from 2 to 10 years of age, data on complete careers were only available for horses born between 1994 and 2001. Data were truncated for horses born between 1986 and 1993, for which data on early performances were missing, and for horses born between 2002 and 2009, for which data on late performances were missing. In order to take selection on performance at 2, 3 and 4 years of age into account, only horses born after 1994 were considered. Horses born after 2008 were also excluded because of the lack of data on performance at 3 years of age. In summary, all performances of horses born between 1994 and 2008 were included in the study, although the performances of some of the horses born between 2001 and 2008 were truncated. However, this was accounted for by introducing a birth year effect in the analysis model. Thus, the dataset consisted of 173 176 born French trotters, 64 274 qualified trotters (37.1%), of which 60 873 entered a harness race and 58 145 won at least 1€ of prize money, and 31 882 entered a race under saddle, of which 25 778 won at least 1€ prize money.

Based on a literature review by Thiruvenkadan *et al*. [4], several traits related to racing data were defined:

- Qualification status (Q), which is obtained once during the horse’s life, and was analyzed as a binary variable (0 = unqualified, 1 = qualified in the qualification test before entering races).
- Age at qualification (days).
- The logarithm of annual earnings divided by the annual number of finished races (i.e. races in which the horse was not disqualified) at 2, 3 and 4 years of age, and the logarithm of the sum of earnings between the age of 5 and 10 years divided by the number of finished races over the same period, calculated separately for harness racing (LnE-H) and racing under saddle (LnE-S) (excluding races under saddle for 2-year-old horses).
- Proportion of finished races (F) without disqualification, treated as a repetitive binary variable for each race started (0 = disqualified, 1 = finished race) at the age of 3 and 4, and between the age of 5 and 10 years for harness racing only.
- Number of races started (S) at 3 and 4 years of age and between the age of 5 and 10 years for harness racing only. A “probit” transformation was applied to the number of races started because its distribution was far from normal.
- Fastest recorded time across all races run for covering a kilometer, in seconds.

Descriptive statistics for most of these traits are in Table 1. Average age at qualification was equal to 1009.2 days (i.e. 2 years 9 months and 5 days; SD: 186.1 days) and for the 47 240 French trotters born between 1994 and 2008 with a best-recorded time, average best time was equal to 1 min 17 s 93 cs (SD: 2 s 90 cs; minimum: 1 min 4 s 90 cs; maximum: 1 min 29 s 90 cs, with cs for centisecond).

**Table 1:** **Descriptive statistics of racing performance traits for trotters born between 1994 and 2008 that entered races between 1996 and 2011**

	**Number of horses**				**Mean**				**Standard deviation**			
**Trait** ^**1**^	**2 y** ^**3**^	**3 y** ^**3**^	**4 y** ^**3**^	**5-10 y** ^**3**^	**2 y** ^**3**^	**3 y** ^**3**^	**4 y** ^**3**^	**5-10 y** ^**3**^	**2 y** ^**3**^	**3 y** ^**3**^	**4 y** ^**3**^	**5-10 y** ^**3**^
*Harness races*												
S	9994	51732	50668	32151	2.87	6.66	9.22	24.46	2.17	4.36	5.62	24.40
F	9994	51732	50668	32151	69%	68%	68%	73%	36%	29%	28%	25%
E, €	9994	51732	50668	32151	4117	7505	9843	29240	9990	17449	17592	67546
LnE-H^2^	7225	42659	40811	26149	6.81	6.74	6.68	6.49	1.26	1.23	1.26	1.27
*Racing under saddle*												
S	218	13108	18709	18944	1.1	3.16	3.57	7.44	0.30	2.84	3.36	10.37
F	218	13108	18709	18944	63%	57%	58%	62%	47%	39%	39%	35%
E, €	218	13108	18709	18944	1103	6175	5888	13380	1882	20931	18909	45937
LnE-H^2^	119	7779	10665	11725	6.83	7.08	6.92	6.71	1.32	1.40	1.42	1.43

^1^Trait abbreviations: S = number of starts, F = proportion of finished races, E, € = earnings, LnE-H = Log(E/(FS)) logarithm of earnings per finished race for harness racing; ^2^for horses with earnings; ^3^y = age in years.

### Horses

Blood samples used in this study were collected previously for a GWAS study on osteochondrosis in French trotters [5]. Animals included in the osteochondrosis study were recruited at CIRALE, the French Imaging and Research Center on Equine Locomotor Disorders, and at a few veterinary clinics from 2008 to 2010. The data comprised 682 genotyped horses. For the current study, 630 horses were retained based on birth year because performances were only available for horses born between 1996 and 2008. Among the 630 horses included in our analysis, 496 (79%) were qualified, which is a much higher proportion than in the overall French trotter population during this time period (37%) and average earnings per finished race were approximately three-quarters of a phenotypic standard deviation higher than the average of the overall French trotter population. This superiority is partly due to the presence of 61 sires, which were selected for breeding based on their own performance. Regarding harness versus under saddle races, the proportion of genotyped horses with earnings for racing under saddle was similar to that in the overall population (27% vs. 26% at 3 years of age, and 41% vs. 45% at 5 years or more). Descriptive statistics of the performances recorded for genotyped horses are in Table 2.

**Table 2:** **Number of genotyped horses with performances**

**Traits/Age**	**TT**	**CT**	**CC**	**Total**
*Number of horses with qualification status*				
	355	245	30	630
*Number of horses with starts/with earnings in harness races*				
2 years	82/72	26/21	0/0	108/93
3 years	261/249	156/130	10/6	427/385
4 years	221/203	163/126	11/9	395/338
5-10 years	143/129	111/101	8/7	262/237
*Number of horses with starts/with earnings in races under saddle*				
3 years	35/18	57/46	4/3	96/67
4 years	58/27	81/59	8/7	147/93
5-10 years	60/30	79/59	8/7	14796

### Genotyping

The genotypes were obtained using the Equine SNP50 BeadChip supplied by Illumina. This chip includes the single nucleotide polymorphism (SNP) *BIEC2-620109* on chromosome 23 at position chr23:22967656 bp that was used to map the *DMRT3* gene by GWAS [1]. This SNP is in strong linkage disequilibrium (LD) with the mutation identified on *DMRT3* (chr 23: 22999655 bp); a LD level (*r*^2^) of 0.91 was recently estimated in a population of 2749 horses from 141 breeds [2]. In our study, the effect of the *DMRT3* gene was analyzed based on the genotypes at SNP *BIEC2-620109*, since its C and T alleles correlate to the phenotypes of the C and A alleles of the *DMRT3* mutation, respectively.

The frequency of the C allele at SNP *BIEC2-62010*9 was 24.2% for the 630 genotyped horses. The frequencies for the three genotypes were 56% (355) for TT, 39% (245) for TC and 5% (30) for CC, which did not deviate significantly from Hardy-Weinberg equilibrium. Most of the 630 horses were born between 2002 and 2008 (87%) and 41% were females. The frequency of allele C did not depend on year of birth, except for a significant drop observed in 2008 (frequency of 15%).

A GWAS was also carried out using the genotypes for all SNPs on the chip. After applying the following quality control criteria i.e. a minor allele frequency greater than 1%, genotype assignment rate greater than 80%, and P-value for the Hardy-Weinberg test greater than 10^−8^, 41 711 SNPs on autosomes were retained.

### Methods to estimate effects of the *DMRT3* gene

First, traits were studied by classical quantitative genetic analysis using all available performance data to determine fixed environmental effects and estimate heritabilities and genetic correlations. Then, the performances corrected for fixed effects were used in a mixed linear model on the genotyped sample in order to determine the effect of the genotype at SNP *BIEC2-62010*9, taking into account the polygenic effect. This model, called FASTA and proposed by [6], was best suited to our situation to reduce type I errors without loss of power [7].

The model used to analyze the performances of the entire dataset was:

1 $$ \mathbf{y}=\mathbf{X}\mathbf{b}+\mathbf{Z}\mathbf{a}+\mathbf{Z}\mathbf{p}+\mathbf{e} $$

For continuous traits, **y** is the vector of performances while for traits with binary variables (qualification and disqualification), **y** is the vector of performances that underlie the trait, since a threshold model was used [8]. **b** is the vector of fixed effects of the birth cohort, namely the combination of the gender at birth (male or female) and of the year of birth (from 1994 to 2008) and thus comprises 30 levels. Two additional fixed effects were added for the best-recorded time i.e. the hippodrome where the races took place (Vincennes, approved hippodromes or other hippodromes) and age at which the best time was recorded (between 2 and 10 years of age). **a** is the vector of random polygenic values (394 367 horses including ancestors), **p** the vector of common environmental effects for different performances of the same horse (only for repeated performances such as disqualification per race), and **e** is the vector of residuals. The variance-covariance matrices of the random effects were $V\left(\mathbf{a}\right)=\mathbf{A}{\sigma}_a^2$, where **A** is the relationship matrix, $V\left(\mathbf{p}\right)=\mathbf{I}{\sigma}_p^2$ and $V\left(\mathbf{e}\right)=\mathbf{I}{\sigma}_e^2$, respectively.

When a multi-trait analysis was performed, the variance-covariance matrices of random effects were the Kronecker product of the covariance matrix between traits and the relationship matrix for genetic effects, and of the identity matrix for residuals, as shown below for two traits:

$$ \begin{array}{c}V\left[\begin{array}{c}\hfill {\mathbf{a}}_1\hfill \\ {}\hfill {\mathbf{a}}_2\hfill \end{array}\right]=\left[\begin{array}{cc}\hfill {\sigma}_{a_1}^2\hfill & \hfill {\sigma}_{a_{12}}\hfill \\ {}\hfill {\sigma}_{a_{12}}\hfill & \hfill {\sigma}_{a_2}^2\hfill \end{array}\right]\otimes \mathbf{A},\\ {}V\left[\begin{array}{c}\hfill {\mathbf{p}}_1\hfill \\ {}\hfill {\mathbf{p}}_2\hfill \end{array}\right]=\left[\begin{array}{cc}\hfill {\sigma}_{p_1}^2\hfill & \hfill {\sigma}_{p_{12}}\hfill \\ {}\hfill {\sigma}_{p_{12}}\hfill & \hfill {\sigma}_{p_2}^2\hfill \end{array}\right]\otimes \mathbf{I},\\ {}V\left[\begin{array}{c}\hfill {\mathbf{e}}_1\hfill \\ {}\hfill {\mathbf{e}}_2\hfill \end{array}\right]=\left[\begin{array}{cc}\hfill {\sigma}_{e_1}^2\hfill & \hfill {\sigma}_{e_{12}}\hfill \\ {}\hfill {\sigma}_{e_{12}}\hfill & \hfill {\sigma}_{e_2}^2\hfill \end{array}\right]\otimes \mathbf{I}.\end{array} $$

Residual correlations between qualification and racing performance could not be estimated when the analysis included both traits since only qualified horses took part in races, and thus, they were fixed *a priori*. This choice is not neutral insofar that it affects the values estimated for other parameters (heritabilities and genetic correlations). Two different scenarios were used to measure the robustness of estimates to the assumed residual correlation between qualification and racing performance. In the first scenario, it was hypothesized that the residual correlation between qualification and racing performances at the ages of 3, 4 and 5 years was the same as that observed between racing performances at the age of 2 years and at the ages of 3, 4 or 5 years. This hypothesis was based on the presence of similar environmental effects for all traits measured at a given age and a decrease of the residual correlation that depended only on the time interval between two observed performances. The second scenario hypothesized that the residual correlation was null.

We analyzed the following set of traits:

- Q and LnE-H at ages 2, 3, 4 and 5 years and more.
- Age at qualification (single trait).
- F at ages 2, 3, 4 and 5 years and more.
- F, S and LnE-H at age 3 years.
- F, S and LnE-H at age 4 years.
- F, S and LnE-H at age 5 years and more.
- Q and best-recorded time.
- LnE-H at ages 3, 4 and 5 years or more and LnE-S at ages 3, 4 and 5 years and more.

Parameter estimates were obtained by Markov chain Monte Carlo (MCMC) Gibbs sampling using the TM software [9]. The number of iterations was set to 50 000. Depending on the trait(s) analyzed, the number of burn-in iterations ranged from 18 000 to 36 000. Samples were thinned and pooled every 20 iterations.

The model used to estimate the effect of genotype at the SNP was:

2 $$ \tilde{\mathbf{y}}=\mathbf{1}\mu +\mathbf{W}\boldsymbol{\upbeta } +\mathbf{Z}\mathbf{u}+\boldsymbol{\upvarepsilon} $$

where **ỹ** is the vector of the performances of the genotyped horses adjusted for the estimated fixed effects of model (1) $\widehat{\mathbf{b}}$: $\tilde{\mathbf{y}}=\mathbf{y}-\mathbf{X}\widehat{\mathbf{b}}$ and for repeated traits, **ỹ** is the vector of the mean of adjusted performances, **W** is the incidence matrix for the horse’s genotypes at the SNP, **β** the vector of the effects of the three genotypes (CC, CT and TT), **u** the vector of polygenic effects (5699 horses including ancestors), with $V\left(\mathbf{u}\right)=\mathbf{A}{\sigma}_a^2$ where **A** is the relationship matrix between all horses, $V\left(\boldsymbol{\upvarepsilon} \right)=\mathbf{I}{\sigma}_e^2$ for non-repeated performances and $V\left(\boldsymbol{\upvarepsilon} \right)=\boldsymbol{\Delta} {\sigma}_e^2$ for repeated performances where **Δ** is a diagonal matrix of the terms ${\delta}_{ii}=\frac{1}{n_i}+\frac{r-{h}^2}{1-r}$, where *n*_*i*_ is the number of performances for horse *i*, *h*^2^ the heritability of the trait and *r* its repeatability. The variance components used were those estimated in model (1). For binary traits, adjusted performances were calculated as follows. Although it would have been better to use the same threshold model as that used to estimate the variance components, this approach was discarded in order to take pre-adjusted performances into account. Expected performances were therefore assigned according to the birth cohort (birth year and gender) and then corrected for the fixed effect of the cohort. The expected performances for each birth cohort were:

$E\left({y}_{ij}\Big|1\right)={\widehat{b}}_j+{\overline{\widehat{a}}}_j+{\overline{\widehat{p}}}_j+{\sigma}_y\left[\frac{\varphi \left(\frac{s-\left({\widehat{b}}_j+{\overline{\widehat{a}}}_j+{\overline{\widehat{p}}}_j\right)}{\sigma_y}\right)}{1-\Phi \left(\frac{s-\left({\widehat{b}}_j+{{\overline{\widehat{a}}}_j}_j+{{\overline{\widehat{p}}}_j}_i\right)}{\sigma_y}\right)}\right]$ for a finished race,

$$ E\left({y}_{ij}\Big|0\right)={\widehat{b}}_j+{\overline{\widehat{a}}}_j+{\overline{\widehat{p}}}_j+{\sigma}_y\left[\frac{\varphi \left(\frac{s-\left({\widehat{b}}_j+{\overline{\widehat{a}}}_j+{\overline{\widehat{p}}}_j\right)}{\sigma_y}\right)}{\Phi \left(\frac{s-\left({\widehat{b}}_j+{{\overline{\widehat{a}}}_j}_j+{{\overline{\widehat{p}}}_j}_i\right)}{\sigma_y}\right)}\right] $$ for a disqualification,

where *y*_*ij*_ is the performance of horse *i* of cohort *j*, ${\overline{\widehat{a}}}_j$ the mean of the estimated polygenic genetic values of the horses of cohort *j*, ${\widehat{b}}_j$ the estimated effect for cohort *j, s* is the estimated threshold, *σ*_*y*_ the standard deviation of underlying performances, *φ* is the density of normal distribution, and Φ is the cumulative normal distribution function. For a repeated performance, ${\overline{\widehat{p}}}_j$ is the mean of estimated permanent environmental values for the horses of cohort *j*. The adjusted performance is ${\tilde{y}}_{ij}\Big|1=E\left({y}_{ij}\Big|1\right)-{\widehat{b}}_j$: for a non-disqualified race, and ${\tilde{y}}_{ij}\Big|0=E\left({y}_{ij}\Big|0\right)-{\widehat{b}}_j$ for a disqualified race. The phenotypic variance used in model (2) was that of model (1), although using expected values alters the variance of adjusted performances. Heritabilities and both genetic and residual correlations were those estimated in model (1). For repeated performances, the residual correlation was that of the permanent environmental effects.

In order to estimate the effect of the genotype at SNP *BIEC2-620109*, performances were analyzed using a multi-trait model (see explanations for the variance-covariance matrices above) with the same groups of traits as those used to calculate the genetic parameters for the entire dataset (listed above), and the same variance-covariance matrices for genetic values and residuals. ASREML software was used [10].

GWAS was also performed for all traits with the remaining 41 710 SNPs, and because of the amount of time required for such a large number of analyses, single-trait models were used. QQ plot analysis was performed to check the distribution of the test statistics. The type I error threshold to detect potential quantitative trait loci (QTL) was set to 5.10^−5^, as in [5] for the same sample (The Wellcome Trust Case Control Consortium, 2007, [11]).

### Modeling the selection process

Two selection processes can be a source of bias in the estimates of the genotype effects at the causal SNP (and not only linked SNP) on racing performances. The first source of bias could come from the fact that a number of wild-type homozygotes (CC) and heterozygotes (which will be shown to both have a negative effect on qualification) were excluded early because these horses did not perform well during initial training. If this selection process occurred before the qualification test, the negative effect of the wild-type allele on qualification may have been underestimated. However, in that case, the mean racing performance of horses with genotype AA remains superior to that of horses with genotype CA, itself superior to that of horses with genotype CC; this ranking of performances according to the three genotypes is identical to that reported by [1]. Thus, this source of bias does not disagree with the results of [1].

Therefore, we focused on the second source of bias which could explain why the surprising result we obtained disagrees with results of [1]: the superiority of heterozygous CA horses compared to mutant homozygous AA horses on late performance. To explain how selection could lead to this result, two traits must be differentiated i.e. (1) the ability to trot easily at an early age, which is assessed during the qualification test and (2) the ability to trot fast and to win races at a mature age, which is assessed by earnings after 4 years of age. The hierarchy of the effects of the different genotypes at the *DMRT3* gene on the first trait is indisputable; this is the effect measured on the “qualification test” trait. To explain a different ranking of the effects of the genotypes on the second trait (measured by earnings after 4 years of age), we need to hypothesize that selection on the first trait will differ according to the racing quality that is expected by the trainer for a horse on the second trait. The hypothesis is that if the horse has difficulties to trot (and thus may be heterozygous CA), the trainer will keep it and train it for qualification only if he/she expects it to be a very good horse for racing when it is older. Selection intensity for horses with genotype CA at an early age is higher than for horses with genotype AA. Among the horses that trot less naturally, only those with the greatest potential enter the qualifying test, whereas practically all horses that trot easily are entered regardless of their racing ability (P. Julienne, personal communication). We tested this hypothesis by modeling the selection process from birth to mature performance. In addition, the French trotter breed is one of the rare horse breeds that is selected for harness racing (trot or pace) and that carries a polymorphism at the *DMRT3* gene [1,2]. Modeling the selection process over generations may explain the polymorphism.

The selection process was modeled using a simplified deterministic model. We assumed that French trotters undergo a 2-step selection process i.e. (1) after qualification (Q) and (2) after mature racing performances (RP), which are considered as two traits. Step (1) was based on a dedicated qualification test and about 40% of the horses born were selected. All sires and 70% of mares must past this step. 30% of mares were not selected. Step (2) was applied on males only and was based on phenotypic racing performance, more precisely on their earnings from 5 years of age. Only 2% of the males born were selected.

Both traits, Q and RP, had a normal distribution and depended on a polygenic value, the effect of the genotype at *BIEC2-620109* and random environmental effects. At each generation *t*, for each genotype *i* (*i* = 1 for genotype CC, 2 for CT and 3 for TT), and each trait *p* (*p* = 1 for trait Q and 2 for trait RP), the mean polygenic values of horses born was denoted *μ*_*tip*_. The frequency of horses born with each genotype *i* at each generation *t* was denoted *f*_*ti*_. The effect of each genotype *i* on each trait *p* was denoted *β*_*ip*_. At each generation *t*, the selection threshold in the first selection step on the first trait was denoted ${\mathit{\mathsf{s}}}_{\mathsf{1},\mathit{\mathsf{t}}}$ and in the second step on the second trait ${\mathit{\mathsf{s}}}_{\mathsf{2},\mathit{\mathsf{t}}}$.

Next, rates of selection of horses with genotype *i* selected in the first (α_1,*ti*_) and second (α_2,*ti*_) selection steps were calculated as follows:

3 $$ {\alpha}_{1,ti}={\displaystyle \underset{-\infty }{\overset{+\infty }{\int }}{\displaystyle \underset{s_{1,t}}{\overset{+\infty }{\int }}{\varphi}_{ti}\left(x,y\right) dxdy}}, $$

and

$$ {\alpha}_{2,ti}={\displaystyle \underset{s_{2,t}}{\overset{+\infty }{\int }}{\displaystyle \underset{s_{1,t}}{\overset{+\infty }{\int }}{\varphi}_{ti}\left(x,y\right) dxdy}}, $$

where *φ*_*ti*_ is the density of the binormal distribution with expectation $$ \left[\begin{array}{cc}\hfill {\beta}_{i1}+{\mu}_{ti1}\hfill & \hfill {\beta}_{i2}+{\mu}_{ti2}\hfill \end{array}\right] $$ and variance matrix **M** = **G** + **R**, where **G** is the variance matrix of polygenic effects between traits 1 and 2, and **R** is the variance matrix of environmental effects between traits 1 and 2. The proportion of horses selected after step (1) was:

4 $$ {\alpha}_1={\displaystyle \sum_{i=1}^3{\alpha}_{1,ti}}{f}_{ti}=0.40 $$

and the proportion of males selected after step (2) was ${\alpha}_{\mathsf{2}}={\displaystyle \sum_{\mathit{\mathsf{i}}=\mathsf{1}}^{\mathsf{3}}{\alpha}_{\mathsf{2},\mathit{\mathsf{t}}\mathit{\mathsf{i}}}}{\mathit{\mathsf{f}}}_{\mathit{\mathsf{t}}\mathit{\mathsf{i}}}=\mathsf{0.02}$. From these equations, thresholds ${\mathit{\mathsf{s}}}_{\mathsf{1},\mathit{\mathsf{t}}}$ and ${\mathit{\mathsf{s}}}_{\mathsf{2},\mathit{\mathsf{t}}}$ were calculated using C05ADF (to locate the zero of a function), D01UAF and D01AMF subroutines (to compute integrals) of the NAG library (Numerical Algorithms Group Ltd., Oxford, UK). Next, the frequency of selected females and the frequency of selected males with each genotype *i* were calculated as ${m}_{ti}=0.7\frac{\alpha_{1,ti}{f}_{ti}}{\alpha_1}+0.3{f}_{ti}$ and ${\mathit{\mathsf{e}}}_{\mathit{\mathsf{t}}\mathit{\mathsf{i}}}=\frac{\alpha_{\mathsf{2},\mathit{\mathsf{t}}\mathit{\mathsf{i}}}{\mathit{\mathsf{f}}}_{\mathit{\mathsf{t}}\mathit{\mathsf{i}}}}{\alpha_{\mathsf{2}}}$, respectively. The polygenic superiority of selected females with genotype *i* for trait *p* was calculated as:

$$ {h}_{tip}=0.7\frac{1}{\alpha_{1,ti}}{\displaystyle \underset{-\infty }{\overset{+\infty }{\int }}z{\displaystyle \underset{-\infty }{\overset{+\infty }{\int }}{\displaystyle \underset{s_{1,t}}{\overset{+\infty }{\int }}{q}_{tip}\left(x,y,z\right) dxdydz}}}, $$

where *q*_*tip*_ is the density of the multi-normal distribution of expectation $$ \left[\begin{array}{ccc}\hfill {\beta}_{i1}+{\mu}_{ti1}\hfill & \hfill {\beta}_{i2}+{\mu}_{ti2}\hfill & \hfill {\mu}_{tip}\hfill \end{array}\right] $$ and variance matrix $$ \left[\begin{array}{cc}\hfill \mathbf{M}\hfill & \hfill \mathbf{G}\left[,p\right]\hfill \\ {}\hfill \mathbf{G}\left[p\right]\hfill & \hfill \mathbf{G}\left[p,p\right]\hfill \end{array}\right] $$. Similarly, the polygenic superiority of selected males was calculated as:

$$ {k}_{tip}=\frac{1}{\alpha_{2,ti}}{\displaystyle \underset{-\infty }{\overset{+\infty }{\int }}z{\displaystyle \underset{s_{2,t}}{\overset{+\infty }{\int }}{\displaystyle \underset{s_{1,t}}{\overset{+\infty }{\int }}{q}_{tip}\left(x,y,z\right) dxdydz}}}. $$

The frequency of each genotype in the next generation was calculated according to Mendelian rules assuming random mating. For example, the frequency of genotype 1 was calculated as:

$$ {f}_{\left(t+1\right)1}={e}_{t1}{m}_{t1}+0.5\left({e}_{t2}{m}_{t1}+{e}_{t1}{m}_{t2}\right)+0.25{e}_{t2}{m}_{t2}. $$

The polygenic superiority of each genotype for horses of the next generation for each trait was calculated assuming additive inheritance. For example, for trait 1 and genotype 1, μ_*(t+*1*)*11_ was calculated as:

$$ \begin{array}{c}{\mu}_{\left(t+1\right)11}={e}_{t1}{m}_{t1}\left({h}_{t11}+{k}_{t11}\right)/2+0.25{e}_{t2}{m}_{t2}\left({h}_{t21}+{k}_{t21}\right)/2\\ {}+0.5\left({e}_{t2}{m}_{t1}\left({h}_{t11}+{k}_{t21}\right)/2+{e}_{t1}{m}_{t2}\left({h}_{t21}+{k}_{t11}\right)/2\right)\end{array} $$

The originality of this breeding scheme lies in the use of a selection threshold in the first selection step that depends on the expected racing quality of the horse for the second trait. We replaced the racing quality expected by the trainer by the phenotypic racing performance obtained in the second selection step (i.e. trait RP), even if this performance was not yet expressed at the time of selection. This assumption is of course very extreme but we used it to obtain an upper bound of the bias due to the potential preferential treatment of some horses at step (1). Thus,

5 $$ {s}_{1,t}=ay+{b}_t, $$

where *y* is the performance of the RP trait as in Equation (3), *a* is a fixed coefficient and *b*_*t*_ was calculated to satisfy Equation (4). We calculated the ratio of the likelihood of being qualified for a horse with RP set at 1 standard deviation from the mean and with mean RP:

$$ \frac{{\displaystyle \underset{a\sqrt{\mathbf{M}\left[2,2\right]}+{b}_t}{\overset{+\infty }{\int }}{\varphi}_{ti}\left(x,\sqrt{\mathbf{M}\left[2,2\right]}\right)dx}}{{\displaystyle \underset{b_t}{\overset{+\infty }{\int }}{\varphi}_{ti}\left(x,0\right)dx}}. $$

This ratio was used to illustrate the value of *a*.

Two results of this selection scheme will be discussed.

First, we calculated the mean value of polygenic and environmental effects on trait RP for horses with different genotypes after the qualification test:

$$ \begin{array}{l}\frac{1}{\alpha_{1,ti}}{\displaystyle \underset{-\infty }{\overset{+\infty }{\int }}z{\displaystyle \underset{-\infty }{\overset{+\infty }{\int }}{\displaystyle \underset{s_{1,t}}{\overset{+\infty }{\int }}{q}_{ti2}\left(x,y,z\right) dxdydz}}},\\ {}\mathrm{and}\\ {}\frac{1}{\alpha_{1,ti}}{\displaystyle \underset{-\infty }{\overset{+\infty }{\int }}z{\displaystyle \underset{-\infty }{\overset{+\infty }{\int }}{\displaystyle \underset{s_{1,t}}{\overset{+\infty }{\int }}{v}_{ti2}\left(x,y,z\right) dxdydz}}},\end{array} $$

where *v*_*tip*_ is the density of the multi-normal distribution of expectation $$ \left[\begin{array}{ccc}\hfill {\beta}_{i1}+{\mu}_{ti1}\hfill & \hfill {\beta}_{i2}+{\mu}_{ti2}\hfill & \hfill 0\hfill \end{array}\right] $$ and variance matrix $$ \left[\begin{array}{cc}\hfill \mathbf{M}\hfill & \hfill \mathbf{R}\left[,p\right]\hfill \\ {}\hfill \mathbf{R}\left[p\right]\hfill & \hfill \mathbf{R}\left[p,p\right]\hfill \end{array}\right] $$.

Second, we calculated the evolution of the frequency of allele C across generations, which was equal to:${f}_{t1}+0.5{f}_{t2}.$

We used the following parameters. Heritabilities for traits Q and RP were equal to 0.56 and 0.26, respectively (i.e. the estimates obtained in the genetic parameters study, as described hereafter). Different genetic (*r*_*g*_) and environmental (*r*_*e*_) correlations between Q and RP that ranged from 0.0 to 0.5 by increments of 0.1 were used to investigate the sensitivity of the results to these parameters, knowing that residual correlations cannot be predicted. The parameter *a* in Equation (5) ranged from 0 (no differential selection on trait Q based on trait RP) to -1 (strong differential selection on Q based on RP) by increments of 0.2. The genotype effect on Q was the estimated effect equal to -0.80 for genotype CC and -0.20 for genotype CT compared to genotype TT (in phenotypic standard deviation unit). The impact of the effects of the genotypes on RP was compared in three scenarios: (i) the effects are as estimated i.e. -0.54 for genotype CC and +0.41 for genotype CT compared to genotype TT (REAL scenario), (ii) genotype does not affect RP (NULL scenario) and (iii) the effect of allele T was assumed to be completely dominant (DOMI scenario). The starting frequency of allele C was 80% and the selection process covered 20 generations. The combination of the hypothesis on *r*_*g*_ (6 cases), *r*_*e*_ (6 cases), parameter *a* (6 cases) and the three REAL, NULL and DOMI scenarios resulted in 648 scenarios.

## Results

### Estimation of genetic parameters

Tables 3, 4, 5, 6, 7, 8 and 9 summarize the genetic parameters obtained for the different traits per set of traits studied. An estimated heritability of 0.30 (standard error, SE = 0.01) was obtained for age of qualification. Generally speaking, heritabilities were higher when qualification was included in the set of traits. Since only the best horses were retained after qualification, the most reliable estimates of heritability for all other performance traits are those that included qualification in the analysis. Estimated heritabilities and genetic correlations were smaller if the residual correlation between the LnE-H and Q traits was set at 0 than if a positive residual correlation was applied (Tables 3 and 4).

**Table 3:** **Genetic parameters for LnE-H and Q**

**Trait**	**LnE-H**				**Q**
	**2 years**	**3 years**	**4 years**	**≥5 years**	
2 years	0.44 (0.02)	0.90 (0.01)	0.76 (0.01)	0.61 (0.03)	0.57 (0.02)
3 years	0.25 (0.02)	0.39 (0.02)	0.91 (0.01)	0.81 (0.01)	0.62 (0.01)
4 years	0.10 (0.02)	0.27 (0.01)	0.27 (0.01)	0.92 (0.01)	0.51 (0.01)
5-10 years	0.11 (0.02)	0.22 (0.01)	0.41 (0.01)	0.29 (0.01)	0.42 (0.03)
Qualification	0.50 (*)	0.27 (*)	0.11 (*)	0.10 (*)	0.55 (0.01)

Heritability (diagonal, (SE)), genetic correlation (upper triangle, (SE)) residual correlation (lower triangle, (SE)), LnE-H: logarithm of earnings per finished race for harness racing, Q: qualification; (*) fixed correlations: residual correlations between Q and LnE-H were fixed to 0.50 at 2 years and closed to residual correlations between LnE-H 2 years and LnE-H 3, 4 and ≥ 5 years for the others.

**Table 4:** **Genetic parameters) for LnE-H and Q**

**Trait**	**LnE-H** ^**1**^				**Q**
	**2 years**	**3 years**	**4 years**	**≥5 years**	
2 years	0.28 (0.01)	0.85 (0.02)	0.76 (0.02)	0.56 (0.02)	0.48 (0.03)
3 years	0.29 (0.01)	0.32 (0.01)	0.91 (0.01)	0.81 (0.02)	0.61 (0.01)
4 years	0.12 (0.02)	0.27 (0.01)	0.25 (0.01)	0.92 (0.01)	0.47 (0.01)
5-10 years	0.14 (0.02)	0.23 (0.01)	0.41 (0.01)	0.26 (0.02)	0.44 (0.02)
Qualification	0.00 (*)	0.00 (*)	0.00 (*)	0.00 (*)	0.56 (0.01)

Heritability (diagonal, (SE)), genetic correlation (upper triangle, (SE)) residual correlation (lower triangle, (SE)), LnE-H: logarithm of earnings per finished race for harness racing, Q: qualification; (*) fixed correlations: residual correlations between Q and LnE-H were fixed to 0.

**Table 5:** **Genetic parameters for F**

**Age**	**2 years**	**3 years**	**4 years**	**≥5 years**
2 years	0.06 (0.00) 0.16 (0.01)	0.75 (0.02)	0.69 (0.02)	0.72 (0.02)
3 years	0.90 (0.01)	0.07 (0.00) 0.18 (0.00)	0.95 (0.00)	0.94 (0.01)
4 years	0.93 (0.00)	0.89 (0.01)	0.08 (0.00) 0.24 (0.00)	0.96 (0.00)
≥5 years	0.73 (0.01)	0.69 (0.01)	0.80 (0.01)	0.09 (0.00) 0.25 (0.00)

Heritability and repeatability (diagonal, (SE)), genetic correlation (upper triangle, (SE)), correlation between permanent environmental effects 1 (lower triangle, (SE)), F: proportion of finished races for harness racing; residual correlations were null by design.

**Table 6:** **Genetic parameters for LnE-H, S and F**

	**LnE-H**	**S**	**F**
*3 years*			
LnE-H	0.31 (0.01)	−0.16 (0.05)	−0.10 (0.05)
S	0.67 (0.05)	0.12 (0.01)	0.21 (0.05)
F	0.32 (0.05)	0.58 (0.04)	0.06 (0.01) 0.18 (0.00).
*4 years*			
LnE-H	0.23 (0.01)	−0.14 (0.06)	−0.22 (0.05)
S	0.85 (0.05)	0.09 (0.01)	0.25 (0.07)
F	0.37 (0.04)	0.64 (0.03)	0.07 (0.01) 0.24 (0.00)
*5-10 years*			
LnE-H	0.25 (0.01)	0.69 (0.03)	−0.19 (0.05)
S	0.93 (0.02)	0.12 (0.01)	0.12 (0.05)
F	0.45 (0.03)	0.65 (0.01)	0.07 (0.01) 0.24 (0.00)

Heritability and repeatability (diagonal, (SE)), genetic correlation (upper triangle, (SE)), correlation between permanent environmental effects (lower triangle, (SE)), LnE-H: logarithm of earnings per finished race for harness racing, S: number of starts, F: proportion of finished races for harness racing.

**Table 7:** **Genetic parameters for LnE-H and LnE-S**

		**Harness racing**			**Racing under saddle**		
		**3 years**	**4 years**	**≥5 years**	**3 years**	**4 years**	**≥5 years**
Harness racing	3 years	0.27 (0.01)	0.93 (0.01)	0.83 (0.01)	0.84 (0.02)	0.82 (0.02)	0.73 (0.02)
	4 years	0.27 (0.01)	0.23 (0.01)	0.93 (0.01)	0.76 (0.06)	0.87 (0.01)	0.82 (0.02)
	≥5 years	0.23 (0.01)	0.41 (0.01)	0.24 (0.01)	0.66 (0.05)	0.80 (0.02)	0.82 (0.03)
Racing under saddle	3 years	0.21 (0.01)	0.07 (0.02)	0.07 (0.02)	0.21 (0.02)	0.81 (0.06)	0.75 (0.04)
	4 years	0.20 (0.01)	0.19 (0.01)	0.10 (0.01)	0.43 (0.02)	0.22 (0.01)	0.85 (0.02)
	≥5 years	0.16 (0.01)	0.25 (0.01)	0.29 (0.02)	0.26 (0.02)	0.43 (0.01)	0.23 (0.02)

Heritability (diagonal, (SE)), genetic correlation (upper triangle, (SE)), residual correlation (lower triangle, (SE)), LnE-H: logarithm of earning per finished race for harness racing, LnE-S: logarithm of earning per finished races for under saddle racing.

**Table 8:** **Genetic parameters for Q and best-recorded time**

	**Qualification**	**Best-recorded time**
*Hypothesis 1*		
Qualification	0.57 (0.01)	−0.67 (0.02)
Best-recorded time	0.00 (*)	0.16 (0.01)
*Hypothesis 2*		
Qualification	0.57 (0.03)	−0.60 (0.02)
Best-recorded time	−0.11 (*)	0.17 (0.02)

Heritability (diagonal, (SE)), genetic correlation (upper triangle, (SE)), residual correlation (lower triangle, (SE)), Q: qualification; (*) fixed correlation, hypothesis 1: residual correlation close to the residual correlation between logarithms of earnings per finished race at 2 and 4 years, hypothesis 2: residual correlation fixed to 0.

**Table 9:** **Genetic parameters for LnE-H and best-recorded time**

	**LnE-S**			**Best-recorded time**
	**3 years**	**4 years**	**≥5 years**	
3 years	0.28 (0.01)	0.94 (0.01)	0.82 (0.02)	−0.92 (0.01)
4 years	0.29 (0.01)	0.24 (0.01)	0.93 (0.01)	−0.88 (0.02)
≥ 5 years	0.27 (0.01)	0.43 (0.01)	0.27 (0.01)	−0.79 (0.02)
Best-recorded time	−0.46 (0.01)	−0.44 (0.01)	−0.43 (0.01)	0.18 (0.01)

Heritability (diagonal, (SE)), genetic correlation (upper triangle, (SE)), residual correlation (lower triangle, (SE)), LnE-H: logarithms of earnings per finished race for harness racing.

For harness racing (Tables 3, 4 and 6), Q was the trait with the highest estimated heritability (0.56), followed by age at qualification (0.30). Estimated heritability of earnings was moderate and stable across age groups (0.26 to 0.31) Disqualification showed low heritabilities (0.06 to 0.09) and average repeatabilities (0.16 to 0.25). The trait “best-recorded time” (Tables 8 and 9) was less heritable (0.17) than earnings and was strongly and favorably correlated with earnings (from -0.79 for earnings at 5 years of age or more to -0.92 for earnings at 3 years of age). For a given year, annual earnings are the product of three traits: annual earnings per finished races (LnE_H), proportion of finished races (F) and number of started races (S). The correlations between these three traits differed according to age group. The genetic correlation between S or F and LnE-H was negative for 3- and 4-year-old horses but positive for horses of 5 years of age or more. All other genetic and environmental correlations were positive. Genetic correlations between LnE-H at 3, 4 or 5 years of age were very high (0.81 to 0.92) and lower between LnE-H at 2 years of age and 3, 4 or 5 years of age (0.56 to 0.85). The genetic correlation between Q and annual earnings was positive but moderate (0.44 to 0.62).

Genetic correlations between LnE-H and LnE-S were high and positive (Table 7) for all age groups (0.82 to 0.87). These correlations decreased with the length of the time interval between performances, and were lowest (0.66) between LnE-H at 5 years of age and LnE-S at 3 years of age. Environmental correlations were moderate, even within a given age group (0.19 to 0.29).

Thus, based on our analysis of traits related to racing performance of trotters, we conclude that racing success has complex characteristics. Qualification had the highest heritability. Genetic correlations between qualification and annual earnings were positive but low. Therefore, qualification and annual earnings are different traits. Annual earnings and ability to finish a race without disqualification are also two different traits, whereas the abilities of a horse to race under harness and under saddle are genetically closely related.

### Genotype effect at SNP *BIEC2-620109*

**Figure 1:**
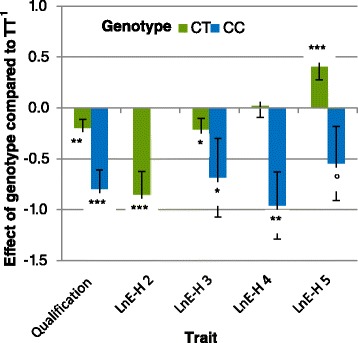
**Effect of the genotype at SNP** ***BIEC2-620109*** **on LnE-H and Q.** LnE-H: logarithm of earnings per finished race for harness racing, Q: qualification; *P* < 0.10, *P < 0.05, **P < 0.01, ***P < 0.001; ^1^in phenotypic standard deviation units.

**Figure 2:**
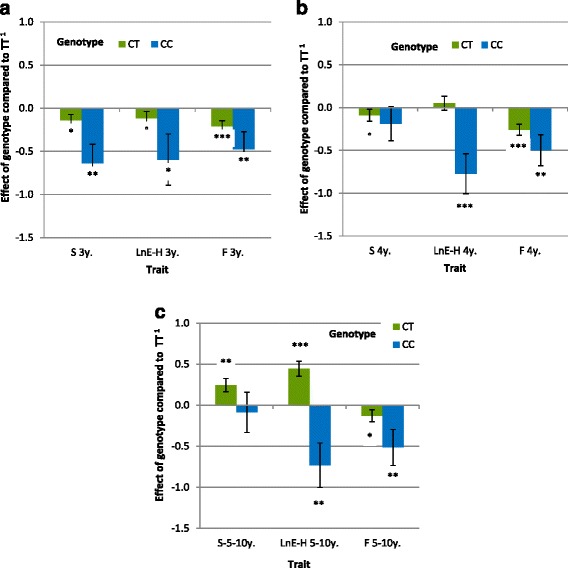
**Effect of the genotype at SNP** ***BIEC2-620109*** **on LnE-H, S and F at 3 years (a), 4 years (b) and from 5 to 10 years of age (c).** LnE-H: logarithm of earnings per finished race for harness racing, S: number of started races, F: proportion of finished races; *P* < 0.10, *P < 0.05, **P < 0.01, ***P < 0.001; ^1^in phenotypic standard deviation units.

**Figure 3:**
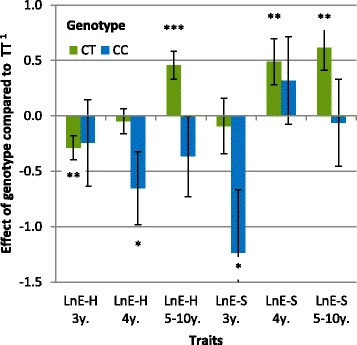
**Effect of the genotype at SNP** ***BIEC2-620109*** **on LnE-H and LnE-S.** LnE-H: logarithm of earnings per finished race for harness racing, LnE-S: logarithm of earnings per finished race under saddle; *P* < 0.10, *P < 0.05, **P < 0.01, ***P < 0.001; ^1^in phenotypic standard deviation units.

Estimated genotype effects are in Figures 1, 2 and 3. The effects were measured in phenotypic standard deviation units (*σ*_*y*_) for all traits. The effect of genotype on best-recorded time was not significant. A significant effect was observed on the age at qualification i.e. 0.88 *σ*_*y*_ for genotype CC and 0.24 *σ*_*y*_ for genotype CT, which is 167 and 45 days later, respectively, than for genotype TT.

Genotype CC had an extremely negative effect on all racing performance criteria, from qualification and earnings to the proportion of finished races, although the effect was not always significant due to the small number of horses with racing performances that carried genotype CC (i.e. 30 horses for qualification status to a maximum of 11 for starts in harness races and only eight for racing under saddle depending on age). The strongest effect (Figure 1, *P* = 9.10^−6^) was the negative effect on qualification i.e. -0.80 *σ*_*y*_, which means that the likelihood for a horse with genotype CC born in 2008 to be qualified was only 20% compared to 48% for a horse with genotype TT. The expected age at qualification for horses with genotype CC was increased by 5.5 months compared with horses with genotype TT *(P* = 1×10^−4^). The genotype effect on earnings or proportion of finished races was less significant (Figure 2). A decrease in LnE-H of 0.6 to 0.8 *σ*_*y*_ was observed between 3 and 5 years of age or more (*P* ranged from 0.0226 to 0.0005). For proportion of finished races, the effect was ½ *σ*_*y*_, which means that the likelihood for a 4-year-old male horse with genotype CC born in 2006 to finish a race was 56% vs. 65% for a horse with genotype CT and 74% for a horse with genotype TT. In racing under saddle (Figure 3), LnE-S was significantly lower (*P* = 0.015) for horses with genotype CC than with genotypes CT or TT at 3 years of age although this effect was not maintained at the age of 4 or 5 years for which no significant difference was observed. Best-recorded times for horses with genotype CC were 1.06 s higher than those of horses with genotype TT, but this difference was not significant (*P* = 0.09).

The effect of allele C was not strictly additive and over-dominance was observed for some traits (when the performance of the heterozygote is higher than the performance of either homozygote). Genotype CT had a negative effect on the likelihood of qualification (*P* = 0.009), which corresponds to a probability of 40% for a male born in 2006 to be qualified if it carried genotype CT vs. 48% if it carried genotype TT. However, this effect was much weaker than for horses with genotype CC i.e. -0.20 *σ*_*y*_ vs. -0.80 *σ*_*y*_ respectively. The age at qualification of horses with genotype CT increased by 1.5 months. Early annual earnings were also impacted with lower LnE-H at 2 years of age and, to a lesser extent, at 3 years of age. However, at 4 years of age, all these differences disappeared, and at 5 years of age or more, the LnE-H of horses with genotype CT was higher than for horses with genotype TT (+0.41 *σ*_*y*_, *P* = 0.0007). By contrast, the likelihood of being disqualified remained higher for horses with genotype CT than for horses with genotype TT, regardless of their age (35% of disqualified races at 3 years of age (*P* = 0.0003) and 27% at 5 years of age or more (*P* = 0.037)) for genotype CT versus 28% and 23% for genotype TT, respectively; the male horses born in 2006 being used as reference. The median number of started races for the whole population was 16, and 22 for horses with genotype CT who had longer careers (more than six months longer) although they entered fewer races at 3 and 4 years of age. For racing under saddle, an increase in annual earnings was observed at 4 years of age and was greater than the effect observed for harness racing at 5 years of age (LnE-S: +0.61 *σ*_*y*_; *P* = 0.0013). No significant effect was observed for the best-recorded time.

### Modeling the selection process

**Figure 4:**
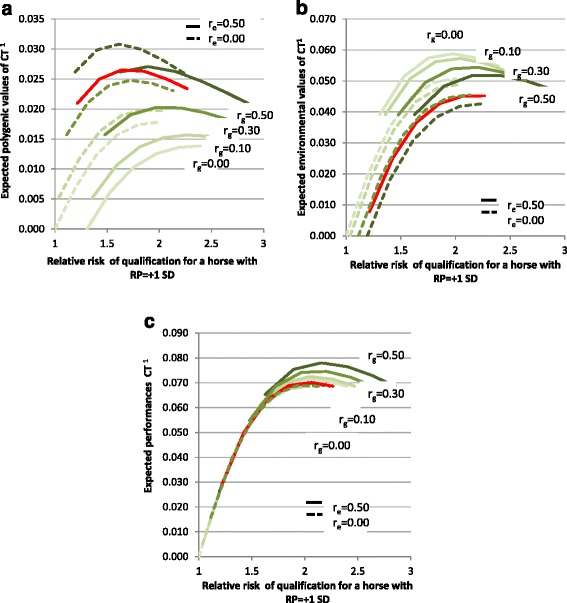
**Expected polygenic values (a), environmental values (b) and summed environmental and polygenic values (c) for RP after selection on Q.** For horses with genotype CT at SNP *BIEC2-620109* when differential selection occurs for qualification test for different assumed future RP*.* RP: racing performances, Q: qualification test, ^1^in standard deviation units, *r*_*g*_ genetic correlation between Q and RP, *r*_*e*_ residual correlation between Q and RP; the red line indicates parameters close to those estimated (r_g_ = 0.44, r_e_ = 0.10).

First, we checked whether the superiority of genotype CT compared to TT for late racing performances was the consequence of a differential selection of the two genotypes at an early age. To answer this question, we compared the polygenic and environmental superiorities for mature racing performance of horses with the three genotypes after the first selection step based on the qualification test. Our results assume that the frequency of allele C is equal to 23.9%. First, let us consider the case for which there was no differential selection according to genotype. The first selection step led to a polygenic superiority of qualified horses compared to unqualified horses for trait Q, which depends on genotype. Because of the negative effect of the genotype on trait Q, the polygenic superiority of horses with genotype CC was higher than that of horses with genotype CT and TT. If the traits are genetically correlated (r_g_ > 0), this difference in polygenic superiority for trait Q would lead to a difference in polygenic superiority for trait RP. Because selection was based on the phenotypic value of Q, the first step of selection also resulted in an environmental superiority of qualified horses for trait Q. For the same reasons, environmental superiority was higher for horses with genotype CC than for horses with genotypes CT and TT. The same applied to the environmental superiority for trait RP if *r*_*e*_ is positive between Q and RP. Among all the scenarios explored, the maximum differences in polygenic and environmental superiorities for trait RP were 0.112 *σ*_*y*_ and 0.168 *σ*_*y*_, respectively for horses with genotype CC and 0.026 *σ*_*y*_ and 0.039 *σ*_*y*_, respectively for horses with genotype CT compared to horses with genotype TT (when *r*_*g*_ = 0.5). Thus, phenotypic RP reached a maximum of 0.280 *σ*_*y*_ for horses with genotype CC and 0.065 *σ*_*y*_ for horses with genotype CT. Results obtained by applying differential selection in the qualification test according to genotype are in Figure 4. Among all scenarios, phenotypic RP never exceeded 0.078 *σ*_*y*_ for horses with genotype CT compared to horses with genotype TT. Such a superiority of CT and CC genotypes on RP significantly deviates from what was observed when estimating genotype effects on RP. Thus, our results suggest that the differences in polygenic and environmental superiorities on trait RP between genotypes that were obtained after the first step of selection based on the qualification test are not sufficient to explain the observed differences in RP between genotypes. Thus, it is necessary to assume a direct effect of the genotype. For horses with genotype CT, the polygenic and environmental superiority due to selection only amounted to 19% (at maximum) of the estimated effect of genotype on LnE-H at 5 years: 0.078 *σ*_*y*_ vs. 0.405 *σ*_*y*_, which led us to assume a direct positive effect of genotype CT, although less strong than its raw estimate from GWAS. To conclude, the estimated positive effect of genotype CT compared to genotype TT for racing performances may be overestimated due to the selection process, but only by 19% at the most.

**Figure 5:**
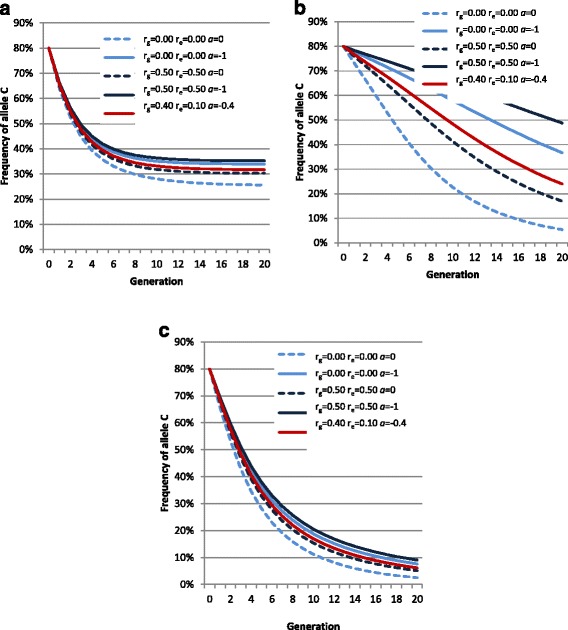
**Variation of the frequency of allele C as a function of the number of selection generations. (a)** Effect of the genotype on RP is as estimated (REAL scenario). **(b)** Effect of the genotype on RP is null (NULL scenario). **(c)** Effect of the genotype on RP is as T is dominant (DOMI scenario). Effects are given with different hypotheses for the genetic (*r*_*g*_) and residual (*r*_*e*_) correlations between qualification (Q) and racing performances (RP). *a*: the threshold for selection on qualification is the linear function (*aRP+ b*).

Our second aim was to explain the high frequency of allele C in the French trotter population after many generations of selection for racing performance. The evolution of the frequency of allele C varies greatly among the three scenarios with different effects of the genotypes on racing performances. In the REAL scenario (Figure 5a) for which the effects of the genotypes on racing performance are the estimated effects, the frequency of allele C decreased with increasing generations and reached an asymptote with a frequency between 25.6% and 35.2% depending on the hypotheses made on *r*_*g*_*, r*_*e*_ and the amplitude of differential selection. This scenario explains why SNP *BIEC2-62010* is still polymorphic in the French trotter population. Based on estimated genetic parameters of 0.44 for *r*_*g*_ and 0.10 for *r*_*e*_, no differential selection and a genotype effect on racing performances that is 19% lower than the estimated effect, the asymptotic frequency of allele C was equal to 23.8%. In the NULL scenario (Figure 5b) for which the *DMRT3* genotype had no effect on racing performance, the frequency of allele C decreased steadily and reached 5.4% after 20 generations when *r*_*g*_ = 0 and *r*_*e*_ = 0 but it was still equal to 48.8% when *r*_*g*_ = 0.50 and *r*_*e*_ = 0.50. The rate of decrease depended mainly on the genetic parameters applied but there was no asymptote. In the DOMI scenario (Figure 5c) for which genotypes CT and TT had identical effects on racing performance, the frequency of allele C decreased rapidly. After 20 generations, the frequency of allele C was lower than 10% in all cases and even reached 2.5% in the most extreme case.

### GWAS: effect of other SNPs on the chip

The distribution of test statistics was validated using QQ plots. The regression coefficient between expected test values and observed values was between 0.85 and 1.14 with a mean of 0.99 and SD of 0.08 over all 16 traits; meaning that as expected with a mixed model, no great inflation was detected in the statistical tests. The most significant SNP remains *BIEC2-620109* on disqualification (F at 4 years of age, *P* = 2.10^−8^) and Q (*P* = 1.10^−7^). However, additional QTL were found for these two traits and the other performance traits (Table 10). For Q, QTL were detected on chromosomes 8, 14 and 28 (1.10^−5^ ≤ *P* ≤ 2.10^−5^). For F at different ages (F at 3, 4 or 5 years of age), a region on chromosome 2 between 47.0 Mb and 49.8 Mb and regions on chromosome 9 and 20 were detected. For LnE-H, QTL were identified on chromosomes 16, 17, 18, 29 and on 23 at 55.0 Mb (a location that differs from that of *DMRT3*). The most interesting QTL were associated with several traits. For example, for the traits LnE-S at 4 years of age and Q, a QTL was detected on chromosome 8 between 72.3 and 74.1 Mb that included two linked SNPs: *BIEC2-106245* and *BIEC2-106276* (r^2^ = 0.10). The minor allele of SNP *BIEC2-10624* had a strong unfavorable effect on LnE-S and the major allele of SNP *BIEC2-106276* had a favorable effect on Q. Another example is for best-recorded time and LnE-H at 4 years of age with a favorable effect of the minor allele of SNP *BIEC2-362276* linked (r^2^ = 0.54) to the major allele of SNP *BIEC2-362278* that also had a favorable effect. This region on chromosome 16 (*P* = 3.10^−5^) is also supported by the fact that SNP *BIEC2-362276* had a less significant effect on LnE-H at 3 years of age (not shown in Table 10, *P* = 7.10^−4^). Note that the effect of SNP *BIEC2-620109* linked to the mutation of the *DMRT3* gene on LnE-H at 5 to 10 years of age is less significant (*P* = 7.10^−4^) than that of the other QTL on chromosome 17 (*P* = 2.10^−6^) or 18 (*P* = 4.10^−5^). SNP *BIEC2-620109* that has an effect on LnE-H would not have been found with the threshold retained (5.10^−5^) for the QTL in Table 10.

**Table 10:** **Significant SNPs other than in the region around** ***BIEC2-620109*** **on Q, LnE-H, LnE-S, S, F, time and age (** ***P*** **< 5.10** ^**−5**^ **)**

**Trait**	**Chr** ^**1**^	**SNP**	**Position (bp** ^**2**^ **)**	**Frequency** ^**3**^	**-Log10(P)**	**Effect** ^**4**^
Q	8	BIEC2-106276	74056773	0.34	4.9	−0.26
Q	14	BIEC2-262117	66099082	0.37	4.6	−0.23
		BIEC2-262136	66106449			
Q	28	BIEC2-745221	44244661	0.35	4.7	−0.25
LnE-H 2y^5^	29	BIEC2-755439	16744350	0.10	4.5	−1.56
LnE-H 3y	23	BIEC2-628318	54969962	0.15	4.3	−0.55
LnE-H 4y	16	BIEC2-362278	76006402	0.41	4.6	−0.48
LnE-H 5-10y	17	BIEC2-385277	76798192	0.28	5.7	−0.82
LnE-H 5-10y	18	BIEC2-418278	72766393	0.06	4.4	−1.21
S 3y	2	BIEC2-493588	83539011	0.33	5.8	−0.34
S 5-10y	1	BIEC2-80578	168362635	0.11	4.5	0.49
S 5-10y	3	BIEC2-772520	12213367	0.17	4.6	0.42
S 5-10y	22	BIEC2-589118	24687615	0.16	4.4	0.40
F 3y	2	BIEC2-476953	46961817	0.32	4.4	0.17
F 3y	2	BIEC2-476987	47071573	0.43	4.4	0.16
F 3y	2	BIEC2-476989	47075607	0.25	4.4	0.18
F 3y	2	BIEC2-476959	46965903	0.40	4.7	0.16
F 3y	2	BIEC2-476920	46721741	0.38	4.7	0.17
F 3y	2	BIEC2-476751	45746148	0.33	4.8	0.17
F 3y	2	BIEC2-476958	46965399	0.40	4.8	0.17
F 3y	2	BIEC2-476695	45549021	0.40	5.2	0.18
		BIEC2-477466	49722328			
F 3y	2	BIEC2-477469	49752293	0.29	5.2	0.19
F 3y	20	BIEC2-532618	42431342	0.38	4.4	−0.15
F 3y	20	BIEC2-534441	46250961	0.09	4.4	−0.26
F 4y	20	BIEC2-534441	46250961	0.09	4.4	−0.25
F 5-10y	9	BIEC2-108684	33584755	0.35	4.5	0.16
F 5-10y	9	BIEC2-108682	33576076	0.33	4.7	0.16
LnE-S 4 y	8	BIEC2-106245	72275101	0.33	4.4	−0.95
LnE-S 4 y	8	BIEC2-106247	72331625	0.21	5.2	−1.23
Time	16	BIEC2-362276	76006247	0.42	4.6	−0.65
Age	22	BIEC2-601669	49750045	0.22	4.5	0.15

^1^chromosome, ^2^ base pairs; ^3^of the minor allele, ^4^in phenotypic standard deviation unit, ^5^y = years; Q: qualification, LnE-H: logarithm of earnings per finished harness race, LnE-S: logarithm of earnings per finished race under saddle, S: number of starts in harness racing, F: proportion of finished harness races, Time: best-recorded time, Age: age at qualification.

## Discussion

The mutation in the *DMRT3* gene was not genotyped directly for any of the horses because we used data from a previous genotyping study without the possibility to re-use blood samples or to genotype other horses. The LD level between SNP *BIEC2-620109* and the *DMRT3* mutation was not reported by Andersson *et al*. [1] but was believed to be very high since the gene was identified with a *P* of 1.7×10^−9^ in a small sample of 70 Icelandic horses using this SNP. Moreover, the frequency of allele C at the SNP in our sample and the frequency of the *DMRT3* mutation in the French trotter sample in Andersson *et al.* [1] were consistent (24% and 23%, respectively). Our results clearly demonstrate that the genotype at SNP *BIEC2-620109* has an extremely significant effect on qualification and racing performances. Although the effect of the *DMRT3* gene itself might have been under-estimated because of an LD level lower than 1, our conclusions remain consistent. A recent paper reported an LD level of 0.91 in a population including 141 different breeds [2]. If such a level was applied to our breed, the estimated effect of the CC genotype at the *DMRT3* gene on qualification would increase from -0.80 *σ*_*y*_ to -0.84 *σ*_*y*_. The LD level in the French trotter breed was expected to be close to that reported by Promerová *et al.* [2] because they used Swedish Standardbred horses to estimate haplotype frequencies for the *DMRT3_Ser301STOP* mutation and SNP *BIEC2-620109* ([2], Table S1). Swedish Standardbred has been developed largely from American Standardbred but with the import of French Trotters [1]. Moreover, Promerová *et al.* [2] argued that the same evolutionary scenario is found for each breed with haplotype TC, which is the ancestral haplotype from which the *DMRT3* nonsense mutation arose and that the frequency of haplotype TA increased because of strong positive selection for the gait mutation.

We measured the effect of genotype CC on the criteria used for trot racing performances in France and demonstrated that it is extremely negative for all criteria (qualification, annual earnings, proportion of finished races) both for harness and under saddle races. The likelihood for a horse with genotype CC to be qualified is divided by 2 compared with other genotypes, and based on their later performances, they are among the 20% worst performers of their generation. This was suggested in [1] based on the fact that allele A was fixed in gaited breeds but was not demonstrated because of an insufficient number of homozygous horses to estimate the effect.

Although of lesser importance, we also demonstrated the negative effect of genotype CT on qualification and early performances. The negative impact of allele C is not additive. The probability for a horse with genotype CT to be qualified was reduced by just 8% (40% vs. 48%) compared to horses with genotype TT. Similarly, annual earnings per finished race were reduced by 1/5 phenotypic standard deviation at 3 years of age for horses with genotype CT. Heterozygous carriers of allele C were also subject to more disqualifications (all age groups), but again the difference is limited with a 9% increase in disqualified races at 4 years of age and a 4% increase at 5 years of age or more. It should be noted that, for this criterion, we could not distinguish between disqualification for galloping (or irregular trotting, the most common error) or ambling. The *DMRT3* mutation affects mainly ambling ability, which suggests that the ability to perform this gait may also be a factor of disqualification in trotting races. Based on earnings for mature horses (≥5 years of age), or even for horses from 4 years of age for races under saddle, heterozygous horses showed higher earnings per finished race and entered more races despite a higher rate of disqualification; their overall earnings were therefore greater (+0.32 *σ*_*y*_, result not shown). The finding that this is a true genetic effect of the horse’s genotype is corroborated by the absence of any other possible explanation such as a selection strategy which would advantage these horses at some point of their career. The measured effect, between 0.3 and 0.4 *σ*_*y*_ (overestimated by 19% at most) explained why allele C has been retained in French Trotters since they were selected during the 20^th^ century. Andersson *et al.* [1] reported a strong and highly significant negative effect for the heterozygous CT horses in their study (n = 17) which differs from our results. This difference between French and Swedish trotters may be due to genetic background (epistasis, role of the other QTL found) and/or genotype x environment interactions. In Sweden, the horses that enter races are less mature than in France and racing under saddle is much less common (a distinctive feature of French trotting races). The specificity of each national racing program may result in differences in the trait, which was measured by earnings. Nevertheless, the mutation in the *DMRT3* gene certainly leads to a more spontaneous ability for lateral gaits (ambling and trotting), and therefore easier qualification, earlier racing and less breaking from gait. The major effect of this gene undoubtedly explains in part the high heritability observed for both qualification (0.56) and annual earnings at 2 years of age (0.30 to 0.40), but once the trotting gait is properly assimilated by the horses, their ability to trot faster forms a distinct trait. In fact, the genetic correlation between qualification and earnings after 5 years of age never exceeded 0.44 and was significantly different from 1, and thus these two traits were never expected to be one and the same trait. The speed trait was more favorably expressed in CT heterozygous horses, especially in racing under saddle where the jockey can probably control breaking. Several QTL that were detected specifically for earnings in harness racing from 3 years to 4 years of age and other QTL for earnings in harness racing after 5 years of age should play a more important role than *DMRT3*.

The implications of this distinctive feature of French trotting races for practical breeding are not straightforward. Before taking action to select against the C allele, it will be necessary to understand the biological mechanism that underlies the advantage observed in heterozygous horses: is the advantage really due to faster movements or does slightly less coordinated gait favor acceleration? Can positive and negative effects be separated? What role is played by the other genes potentially identified by GWAS, and how do they interact with *DMRT3*? Only studies on the gait of horses with various genotypes will answer these questions. Moreover, breeders will need to simultaneously manage two objectives: (i) eliminating foals with genotype CC and (ii) maintaining heterozygous CT horses in the population.

## Conclusions

The identification of the *DMRT3* gene and its major effect was a great achievement in the field of horse breeding and racing. The results published by the Swedish team demonstrated beyond doubt its crucial role in the spinal cord neuronal circuits that control stride in mammals. The mutant A allele has been selected over the C allele in the major trotting breeds because of its essential role in ambling. It was therefore surprising to discover that there is still allelic variation at this locus (e.g. that it has not been fixed by selection) in a breed such as the French Trotter that has been selected for more than a century on racing performances. Based on the genotypes of 630 French Trotters at a SNP associated with the *DMRT3* mutation, we showed that all traits associated with racing were negatively affected in homozygous CC horses but that this was not the case for heterozygous CT horses (with genotype CA at the *DMRT3* gene). Heterozygous CT horses performed slightly less well in the qualification race but, in the end, achieved better results than homozygous TT horses (with genotype AA at the *DMRT3* gene) in the French racing circuit which has the specificity of being based on the performances of more mature horses and racing under saddle. This superiority of heterozygous CT horses most probably explains why this polymorphism still exists in French Trotters. More work is needed to determine the biological and neurological pathways that underlie this advantage and the relationships between *DMRT3* and other trot-related genes, and to draw up a mating and selection design that benefits from these results.
